# Comparison of collapsing methods for the statistical analysis of rare variants

**DOI:** 10.1186/1753-6561-5-S9-S115

**Published:** 2011-11-29

**Authors:** Carmen Dering, Andreas Ziegler, Inke R König, Claudia Hemmelmann

**Affiliations:** 1 Institut für Medizinische Biometrie und Statistik, Universität zu Lübeck, Universitätsklinikum Schleswig-Holstein, Campus Lübeck, Maria-Goeppert-Str. 1, 23562 Lübeck, Germany

## Abstract

Novel technologies allow sequencing of whole genomes and are considered as an emerging approach for the identification of rare disease-associated variants. Recent studies have shown that multiple rare variants can explain a particular proportion of the genetic basis for disease. Following this assumption, we compare five collapsing approaches to test for groupwise association with disease status, using simulated data provided by Genetic Analysis Workshop 17 (GAW17). Variants are collapsed in different scenarios per gene according to different minor allele frequency (MAF) thresholds and their functionality. For comparing the different approaches, we consider the family-wise error rate and the power. Most of the methods could maintain the nominal type I error levels well for small MAF thresholds, but the power was generally low. Although the methods considered in this report are common approaches for analyzing rare variants, they performed poorly with respect to the simulated disease phenotype in the GAW17 data set.

## Background

New technologies allow the sequencing of genomes of a large number of individuals, thus identifying millions of rare variants in the genome. This allows researchers to investigate the common disease/rare variants (CDRV) hypothesis. Because these variants are rare or are even private mutations, standard statistical approaches fail. However, different variants within a gene may act similarly. Therefore one approach of making data accessible to statistical analysis is pooling rare variants in a specific genetic region of interest [1]. Another advantage of this collapsing approach is that investigations can be focused on causal relations between genes and the phenotype of interest.

In this study we focus on the CDRV hypothesis by grouping multiple rare variants according to a gene. To test for groupwise association with the simulated disease status in unrelated individuals, we compare the following five collapsing approaches: cohort allelic sum test (CAST) [2], combined multivariate and collapsing (CMC) method [3], weighted-sum (WS) statistic [4], and two rare variant tests (RVT1, the test with proportion coding; and RVT2, the test with indicator coding) [5]. To this end, we use the simulated data provided by Genetic Analysis Workshop 17 (GAW17).

## Methods

The five different collapsing approaches have been described in detail elsewhere, for example, by Dering et al. [1]. Here, we briefly describe the specific configurations used in our study.

For CAST we use the Fisher two-sided exact test for statistical analysis, as suggested in the original publication [4]. For the CMC method, we use the Fisher product method as the multivariate test.

The WS statistic was proposed by Madsen and Browning [4]. In the WS approach, *p*-values are obtained by permutation. The classical permutation approach estimates the proportion of permuted test statistics that exceeds the original test statistic. If *p*-values are supposed to be low, a large number of permutations is required, and this requires a lengthy computing time. Therefore Madsen and Browning proposed to estimate the first two moments of the permutation distribution under the null hypothesis of no association in the first step. In the second step, the estimated mean and standard deviation from the permutation distribution under the null hypothesis are used to standardize the original test statistic. This standardized test statistic is finally used to estimate the *p*-value from the standard normal distribution; for details, see Dering et al. [1] and Madsen and Browning [4]. Here, we fix the number of permutations to 1,000, as suggested by Madsen and Browning [4].

### General data information

The GAW17 sequence data are based on the 1000 Genomes Project [6], and phenotypes were simulated by the GAW17 team [7]. We consider the collection of 697 unrelated individuals with a simulated disease phenotype. The simulated affection status for a common disease (frequency of 30%) was provided for all 200 replicates of the phenotype and 24,487 single-nucleotide polymorphisms (SNPs) in 3,205 genes, all of which were autosomal. Allele frequencies for the markers range from private variants to minor allele frequencies (MAFs) greater than 0.4, with most variants being rare (more than 50% had MAF < 0.01, and only 10% had MAF > 0.05).

### Data preprocessing

To compare the different collapsing approaches, we consider the binary disease phenotype without any covariates. We first convert the genotype files of the GAW17 data using PLINK, version 1.05 [8,9], to generate additive SNP coding. For our analyses we implement CAST [2], the CMC method [3], and the WS method [4] in R, version 2.12.0 [10]. The input data are further converted with GTOOL, version 0.5.0 [11], into the SNPTEST format for running GRANVIL, version 0.4, which is an implementation of RVT1 [12], and for running CCRaVAT [13], an implementation of RVT2 [5]. GRANVIL and CCRaVAT are run with default values.

Only genes with at least two variants are included. Furthermore, we exclude the 695 spuriously associated genes that were identified by Luedtke et al. [14].

We investigate three groups of variants: (1) all variants, (2) synonymous variants only, and (3) nonsynonymous variants only. Because the effect size of variants may depend on their frequency, we consider different MAF limits for collapsing: 0.01, 0.02, 0.03, 0.05, 0.07, and 0.10. For example, for synonymous variants with MAF < 0.01, only 4,218 variants in 765 genes with at least 2 variants remained.

For the CMC method in all scenarios we collapse variants with MAF < 0.01, and variants with larger MAFs were investigated separately for each gene. As a consequence, in scenarios with MAF < 0.01, the CMC method and CAST were identical.

For controlling the family-wise error rate (FWER), which is the probability of committing at least one type I error, we set the multiple significance level to 0.05. The local significance levels are obtained by Bonferroni correction, that is, by dividing the multiple level by the number of genes in the individual scenarios under the assumption of independent genes. In the previous example with synonymous variants and MAF < 0.01, the local significance level is therefore 0.05/765 ≈ 6.5 × 10^−5^.

We use two different definitions of power, namely, average and minimal power, to compare the performance of the different collapsing methods. The average power is defined as the expected proportion of identified true gene disease associations among all true associations, and the minimal power is defined as the probability to detect at least one of the true associations. The FWER and the power are averaged by the number of replicates.

## Results

### Type I error

**Figure 1:**
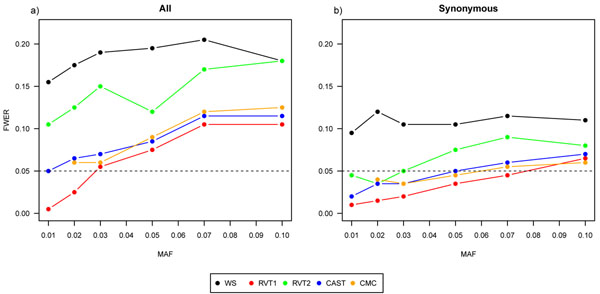
**FWER for five collapsing approaches**. Family-wise error rate (FWER) of all five collapsing approaches depending on different minor allele frequency (MAF) thresholds (a) without restriction to SNP type and (b) with restriction to synonymous SNPs.

None of the considered collapsing methods satisfied the multiple significance level in all scenarios (Figure 1). Without restriction to SNP type, almost none of the methods kept the multiple significance level, except RVT1, the CMC method, and CAST for small MAF limits (Figure 1a). When considering only synonymous SNPs, the FWER was generally smaller, and for small MAF thresholds RVT1, RVT2, CAST, and the CMC method satisfied the multiple significance level (Figure 1b). For nonsynonymous SNPs only, the FWER was in between the FWER of the other two cases (results not shown).

### Power

The minimal power for all methods was low in all scenarios (Table 1), and it ranged from 0% to 28.5%. The minimal power of all methods was 0 for almost all MAF limits when the analysis was restricted to synonymous SNPs. In most of the considered cases, the minimal powers of RVT2, CAST, and the CMC method were identical. The average power was close to 0 for all methods in all scenarios (range, 0–1.2%; results not shown).

**Table 1:** Minimal power of five collapsing approaches

SNP type	MAF	WS	CMC	CAST	RVT1	RVT2
All	0.01	0.015	–	0.010	0.010	0.010
	0.02	0.025	0.030	0.030	0.040	0.030
	0.03	0.070	0.050	0.050	0.060	0.055
	0.05	0.035	0.050	0.050	0.060	0.050
	0.07	0.120	0.115	0.115	0.125	0.115
	0.10	0.135	0.075	0.075	0.115	0.070
Synonymous	0.01	0	–	0	0	0
	0.02	0.005	0	0	0	0
	0.03	0.005	0	0	0	0
	0.05	0	0	0	0	0
	0.07	0.005	0.005	0.005	0	0.005
	0.10	0	0	0	0	0
Nonsynonymous	0.01	0.010	–	0.010	0	0.010
	0.02	0.050	0.045	0.045	0.025	0.045
	0.03	0.135	0.120	0.120	0.090	0.120
	0.05	0.130	0.120	0.120	0.090	0.120
	0.07	0.285	0.170	0.170	0.285	0.170
	0.10	0.235	0.170	0.170	0.285	0.170

Minimal power of all five collapsing approaches for different SNP types and MAF thresholds to control a family-wise error rate ≤ 0.05. WS, weighted-sum method; CMC, combined multivariate and collapsing method; CAST, cohort allelic sum test; RVT1, rare variant test with proportion coding; RVT2, rare variant test with indicator coding.

## Discussion and conclusions

In this study, we focused on the CDRV hypothesis by grouping multiple rare variants according to genes. We compared collapsing approaches proposed in the literature using the GAW17 data of unrelated individuals.

Results differed substantially with respect to SNP functionality. Specifically, for nonsynonymous variants the minimal power was up to 28.5%. In contrast, the minimal power was close to 0 for all methods in almost every scenario when only synonymous variants were considered. However, this result is not surprising because no synonymous variants are directly associated with the simulated disease phenotype.

Furthermore, the minimal power of all methods differed for several MAF thresholds (Table 1). Price et al. [15] proposed an alternative method to overcome the problem of selecting a MAF threshold. They calculated the maximum test statistic over all reasonable MAF thresholds. An interesting approach could therefore be a combination of the maximum test statistic of Price et al. [15] with the different collapsing methods.

Considering only valid methods (i.e., test statistics with FWER ≤ 0.05), the WS approach could not be applied. Furthermore, the remaining methods could be applied only in the scenarios with MAF thresholds smaller than 0.05 with respect to the FWER. If both type I error and power are considered simultaneously, we have to conclude that none of the methods perform sufficiently well on the simulated data.

In conclusion, none of the investigated approaches can be recommended with respect to the simulated disease phenotype. An improvement in the performance of the methods could be achieved if the underlying disease model was known. Furthermore, the inclusion of prior knowledge, such as pathway information, or putative functional information of the variant might be beneficial for the analysis of real data sets.

## Competing interests

The authors declare that there are no competing interests.

## Authors’ contributions

CD implemented the considered methods and performed the statistical analysis. IRK and AZ participated in the design of the study and in the selection of the methods. CH conceived of the study and participated in its design and coordination. All authors drafted, read and approved the final manuscript.
